# Peptides, Peptidomimetics, and Polypeptides from Marine Sources: A Wealth of Natural Sources for Pharmaceutical Applications

**DOI:** 10.3390/md15040124

**Published:** 2017-04-22

**Authors:** Rushikesh Sable, Pravin Parajuli, Seetharama Jois

**Affiliations:** Department of Basic Pharmaceutical Sciences, School of Pharmacy, University of Louisiana at Monroe, 1800 Bienville Drive, Monroe, LA 71201, USA; sablerv@warhawks.ulm.edu (R.S.); parajup1@warhawks.ulm.edu (P.P.)

**Keywords:** marine peptides, antifungal peptides, antimicrobial peptides, extraction of peptides

## Abstract

Nature provides a variety of peptides that are expressed in most living species. Evolutionary pressure and natural selection have created and optimized these peptides to bind to receptors with high affinity. Hence, natural resources provide an abundant chemical space to be explored in peptide-based drug discovery. Marine peptides can be extracted by simple solvent extraction techniques. The advancement of analytical techniques has made it possible to obtain pure peptides from natural resources. Extracted peptides have been evaluated as possible therapeutic agents for a wide range of diseases, including antibacterial, antifungal, antidiabetic and anticancer activity as well as cardiovascular and neurotoxin activity. Although marine resources provide thousands of possible peptides, only a few peptides derived from marine sources have reached the pharmaceutical market. This review focuses on some of the peptides derived from marine sources in the past ten years and gives a brief review of those that are currently in clinical trials or on the market.

## 1. Introduction

Naturally occurring peptides, including hormones, neurotransmitters, anti-infective agents, and growth factors, play a major role in human physiology. From the literature, it is estimated that nearly 7000 peptides/peptidomimetics have been identified from natural resources [1,2,3]. With regard to drug design, peptides serve as effective pharmacophores because of their selectivity and high binding affinity to carry out signaling processes in cells. With 20 different amino acids, there are several functional groups available in peptides. If we consider the variations in possible side chains and possible combinations of backbone and side chains with a minimum of 11–16 amino acid residues, a large number of possible pharmacophores can be generated [4]. Kazanov and Carlson [4] estimate that the total number of valid ligand binding sites in proteins is around 7700, and a tetrapeptide library can be used to generate possible molecules with a pharmacophore that can be used to cover most of these binding sites. Thus, the potential for peptide-based drug design is large. Furthermore, various possible chemical spaces of the peptide (different possible conformations that can be generated by functional groups in a peptide molecule by incorporating 20 amino acids with different side chains) can be put into practical use because of the ease of synthesis of peptides by solid-phase peptide synthesis. Because of their natural ability to bind to protein receptors and the large available chemical space and few off-target effects, peptides can be used to design building blocks that are close in shape to agonist or antagonist binding sites of the receptor. The high degree of specificity of the binding of peptides to proteins is a result of evolutionary selection of complementary functional groups or charges to bind to specific targets among a variety of targets available in proteins that carry out a variety of physiological processes [5]. In many cases, naturally occurring peptides are suitable as starting points for pharmacophores or the design of drug-like molecules with incorporated secondary structural elements [6,7].

Regarding drug-like properties, peptides possess the properties of antibodies and small molecules and, hence, they bridge the gap between antibody drugs and small molecules. They can be made into peptidomimetics with small molecule properties, yet retain some amino acids and binding specificity to maintain characteristics of proteins or biotechnology-based drugs [8]. Peptides have limitations in terms of delivery, as most peptides are not orally available and have a short half-life in vivo due to proteolytic degradation. In addition, most peptides are cleared from the kidneys after intravenous administration [9]. However, these limitations can be overcome by using different design strategies [10,11,12,13] or peptides that are naturally stable against enzymatic and thermal degradation [14,15]. Most peptide drugs do not follow the “rule of five” proposed by Lipinski et al. [16] and modified by Veber et al. [17]. Despite several limitations of peptide-based drugs, there are a number that have made it to the market and, hence, there has been an increased interest in the pharmaceutical industry to bring more peptide-based drugs to the market. Examples of drugs that have reached the $1 billion market include glatiramer acetate (an immunomodulatory drug used to treat multiple sclerosis), leuprolide acetate, goserelin acetate, and octreotide. There are more than 50 FDA-approved, peptide-based drugs on the market, and more than 140 in the pipeline [18,19]. The number of available peptide drugs is very small, around 1.5% of the total drugs on the market. However, growth in peptide-based drugs is seen when we consider the number of peptides entering clinical trials—around 20 per year in the past few years [20].

### Peptides from Natural Sources

Nature provides a variety of peptides that are expressed in most living species. These peptides provide a very good chemical location for exploration, since evolutionary pressure and natural selection have created and optimized these peptides to bind to receptors with high affinity. Hence, natural resources provide an abundance of chemical space to be explored in peptide-based drug discovery. Many peptides that have d-amino acid substitution [21] have been isolated from the skin of frogs and toads [22,23]. More than 300 antimicrobial peptides that work against pathogens have been discovered and characterized [24]. Peptides from the venom of spiders, snails, and snakes have evolved as a means of predators. Some of these are being used to treat neurological or cardiovascular diseases, or to alleviate pain [25]. One of the best examples of such natural peptides is exenatide, a glucagon-like peptide-1 agonist used in the treatment of diabetes mellitus type 2. This peptide was originally discovered in a hormone found in the saliva of the Gila monster. A synthetic version of this peptide is now produced as a commercial drug [26]. Thus, natural resources can be used to explore the chemical space that has evolved for millions of years. Although there are peptides from many different natural resources, in this paper we restrict our review of peptides and peptidomimetics to those originating from marine natural resources. In terms of the size of the peptides, we will limit it to those with less than 50 amino acids.

Marine sources are the largest supply of peptides and natural small molecules, since they represent half of the total global biodiversity. Among evolutionary peptides, marine resources provide a wide variety of chemical space exploration as marine animals live in very different and competitive environments compared to a terrestrial setting. Marine organisms are exposed to hostile environments, filled with microbes that can infect at every opportunity. Hence, the majority of marine organisms produce antimicrobial peptides (AMPs). Because of the vast number of antimicrobial peptides available from marine sources, we will describe only a few of these under each phylum in this review. Antimicrobial peptides are classified based on the amino acids present in the sequence and secondary structure of the peptide. The three classes of antimicrobial peptides are (a) linear peptides with α-helical amphipathic structures; (b) β-sheet or mixed α-helical/β-sheet peptides with disulfide bonds; and (c) peptides with a high content of amino acids Arg, Gly, Pro or Trp, with extended structures. While most AMPs are cationic and insert into the membrane, many anionic peptides have also been reported. They insert into the membrane and destabilize microbial membranes by pore formation. They can also translocate across membranes and/or inhibit metabolic functions of microorganisms [27,28]. AMPs are known to target Gram-positive and Gram-negative bacteria, fungi, and enveloped viruses [29]. Furthermore, they can target cancer cells and stimulate immunity. Recent articles by Liu et al. [30] and Anjun et al. [31] provide reviews of many new AMPs and their possible uses for pharmaceutical purposes.

AMPs can be biosynthetically classified into ribosomally synthesized peptides (RPs) and non-ribosomally synthesized peptides (NRPs). Usually, ribosomal-based synthesized peptides can contain only the 20 unmodified proteinogenic amino acids, while NRP enzymes are able to incorporate more than 100 non-coded non-proteinogenic amino acids. As a result, RPs should have more limited chemical diversity compared to NRPs. However, certain ribosomally synthesized peptides are produced as protein precursors, which are further modified by dedicated enzymes encoded in the peptide gene cluster, thus leading to mature and often highly modified active peptides. These peptides, which have attracted much interest in recent years, also include unusual or modified amino acids in their sequences due to the posttranslational modifications, and are called ribosomally synthesized and posttranslationally modified peptides (RiPPs) [32]. The non-ribosomal peptides are biosynthesized using polyfunctional enzymes called non-ribosomal peptide synthetases (NRPSs) and the related polyketide synthases (PKSs) [33,34], associated in some cases as hybrid NRPS-PKS. Therefore, the evolution of nature has produced chemical diversity through these two different biosynthesis pathways. Some NRPs, such as pristinamycin, actinomycin D, vancomycin, and cyclosporine A, are used for therapeutic purposes [35]. Because of extensive posttranslational/co-translational modifications, these RiPPs can have restricted conformational flexibility that allows them better target recognition. Furthermore, they can acquire increased metabolic and chemical stability, making them suitable for pharmaceutical applications [36]. Some examples of RiPPS are lanthipeptides, including lantibiotics, a class of antimicrobial peptides, which are characterized by the presence of unusual thioether linkages; polytheonamides, a class of cytotoxic peptides obtained from the *Theonella swinhoei* sponge that contain an unusual *N*-acyl moiety; and cyanobactins isolated from the marine tunicate *Lissoclinum patella,* which are cytotoxic peptides characterized by an N-to-C macrocyclization and the presence of diverse heterocycles such as oxazoles and thiazoles [32].

Naturally occurring peptides from marine sources may have modified structures in the backbone or side chain structure compared to peptides in humans because of the aggressive demands of their environment; hence, they are suitable as scaffolds for drug design and provide stability against enzymes and thermal conditions. Because of the nature of the marine environment, these peptides can have a very broad spectrum of activity against different pathogens. The majority of these peptides are obtained from sponges, ascidians, and mollusks. Many peptides are also obtained from seaweeds. Peptides obtained from marine resources that can be used as anti-infective and therapeutic agents are described in some recent review articles [37,38,39]. Marine organisms are also found to live in association with symbiotic bacteria. These bacteria produce several chemicals, including peptides. Symbioses between microorganisms and other higher marine organisms are more predominant in the marine than in the terrestrial environment [40,41]. The association between marine organisms and microorganisms produces a diverse array of chemicals. This symbiosis has created biochemical pathways in marine organisms and bacteria that result in the production of pharmaceutically applicable natural products. Among such marine organisms, sponges harbor microorganisms on their surfaces, in their canal systems, and also in the intercellular matrix comprising up to 40% of their biomass [42].

Many marine eukaryotes establish stable associations with bacterial partners and depend on them for growth, development, acquisition of nutrients, and protection from colonization and predation [43,44]. Such complex symbiotic assemblages are termed “holobionts” [45]. For example, corals form a symbiosis between polyps, unicellular algae (zooxanthelles), and associated microorganisms. Corals also offer surfaces for the growth of microorganisms, which constitutes the mucus surface that covers the coral and prevents colonization by bacteria [46]. Another example is marine microalgae, which are home to a diverse group of bacteria [47]. The microorganisms on marine animal surfaces also produce secondary metabolites to enhance their survival in the conditions present on the surface of the host’s body. These secondary metabolites may be useful as lead compounds in the drug discovery process [48,49,50]. Thus, marine microbial symbionts are a hotspot for drug discovery research in the field of marine natural products. Although a wide variety of unexplored molecules is available in the marine environment, screening key components for pharmaceutical purposes is a difficult task [51,52]. In addition, secondary metabolites derived from marine animals complicate the issue of screening. However, these secondary metabolites also exhibit antibiotic, antiparasitic, antiviral, and anticancer activities [53]. Marine organisms produce toxic peptides, such as toxins from conus, which serve as specific channel blockers and are useful in neurophysiological and neuropharmacological studies [54]. Some of these toxins may be harmful when used directly as therapeutic agents, and they may have to be modified to reduce toxicity. In this review, we cover the general aspects of peptides from marine resources and their isolation procedures as well as structure-activity relationships. Our intention is to provide readers with a review of the work done in the past five to 10 years. The peptides we have covered in this review are from marine sources that are discovered through classical methods, such as direct extraction from the marine source, or by enzymatic methods from natural sources. Peptides from marine resources can be discovered using recently developed genomic approaches. Genomic approaches have diversified the number of marine peptides that exhibit pharmacological activity [55,56,57,58]. An exhaustive list of marine peptides and polypeptides and their pharmacological activities have been provided in some reviews [31,34,37,38,59,60,61,62]. We have included the names of some of the marine peptides derived from marine resources that have been published from 2009 to 2016 in Table 1.

## 2. Isolation and Extraction of Marine Bioactive Peptides (MBPs)

**Figure 1:**
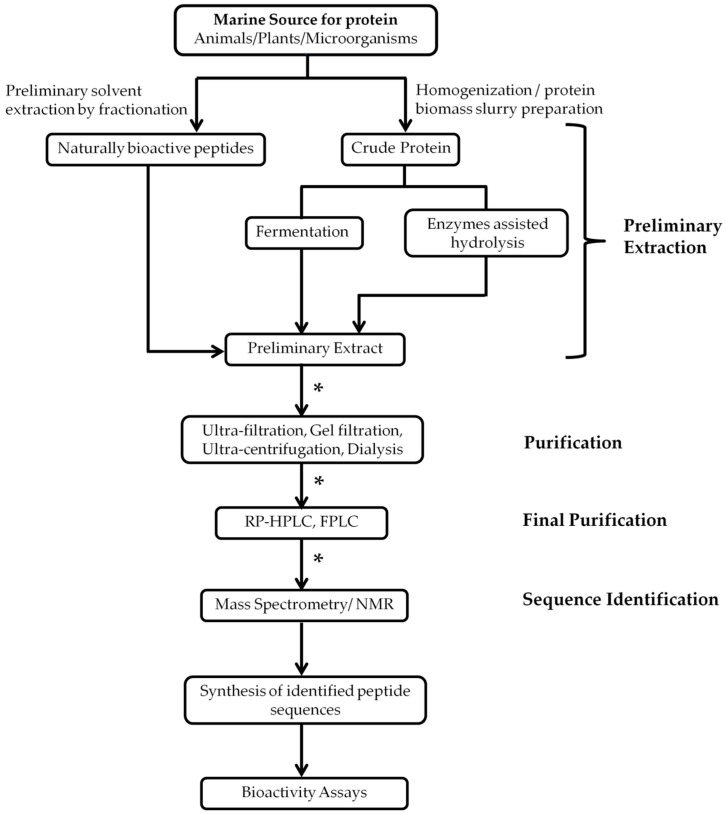
A schematic representation of the general extraction procedure for marine peptides. * Stages where bioassays are performed for screening.

Bioactive peptides can be broadly classified as (A) naturally active peptides that can be directly extracted from marine sources; (B) peptides that can be produced by hydrolysis of parent proteins from marine sources with the use of a variety of enzymes; and (C) peptides that can be obtained from a fermentation product of microorganisms [109]. The extraction procedure for these different classes of bioactive peptides differs at the preliminary extraction phase (Figure 1). The purpose of preliminary extraction is to separate the large proteins from the rest of the particulate biomass. Many different techniques such as ultrafiltration, microfiltration, reverse osmosis, nanofiltration, gel filtration, and ultracentrifugation [110] are also used for the first steps of purification. After the preliminary extraction and separation of peptides, various purification techniques including ion-exchange chromatography, gel permeation, and reversed-phase high-performance chromatography can be used for the next steps to afford pure peptides. Cole et al. [111] performed the separation of cationic peptides obtained from skin secretions of the fish *Pleuronectes americanus* using ion-exchange chromatography along with reversed-phase high pressure liquid chromatography (RP-HPLC). Song et al. [112] used gel permeation chromatography to separate hydrophobic peptides from peptides bearing net charges. Further purification of these peptides obtained from the fish *Setipinna taty* was carried out using RP-HPLC. Advanced mass spectrometric techniques such as Matrix-Assisted Laser Desorption Ionization-Time of Flight Mass Spectrometry (MALDI–TOF–MS/MS) or NMR spectroscopy are some popular techniques for the identification of amino acids and their sequences in peptides [93,94,113].

**Figure 2:**
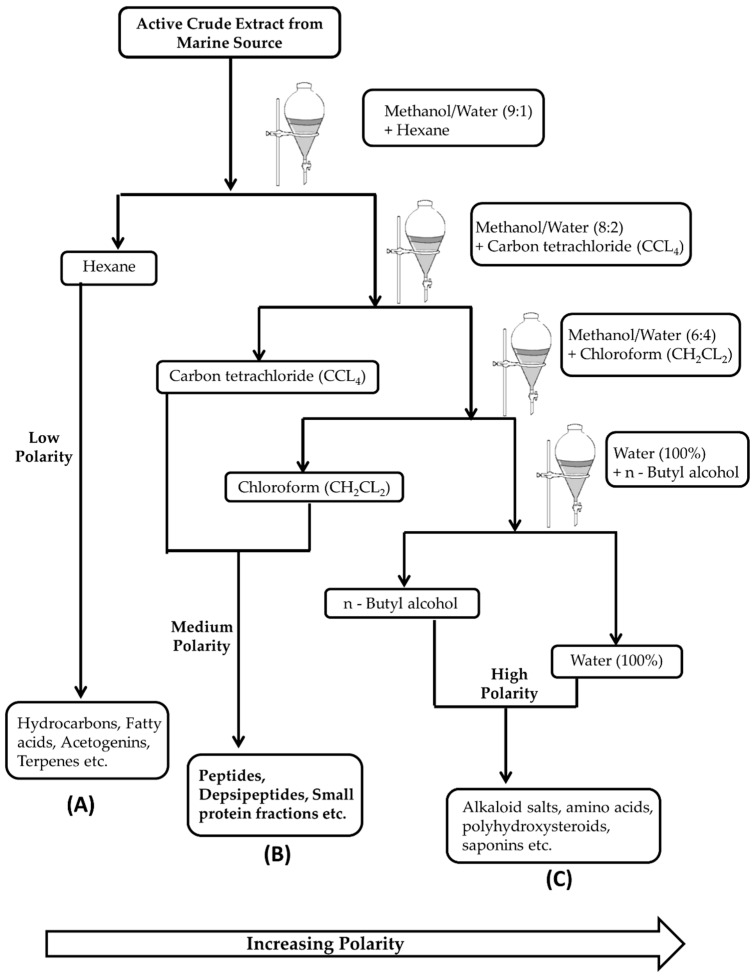
A schematic representation of the solvent gradient extraction procedure of different components from natural marine sources. Adapted from Riguera, R. *J. Mar. Biotechnol.* **1997**, *5*, 187–193.

Naturally, bioactive peptides can be directly extracted from biomass with the appropriate solvent system; the most commonly used solvents are methanol and acetone [114,115]. After this preliminary direct extraction, isolation can be done in several steps. In the fractionation step, the crude extract is exposed to different gradient systems sequentially, from low polarity to high polarity solvents (Figure 2). Usually, a medium polarity gradient (e.g., dichloromethane, tetrahydrofuran, ethyl acetate, etc.) holds the peptides and depsipeptides in the solution, as indicated in Figure 2B [115].

For further purification, these gradient system fractions can usually be separated by normal or reversed-phase high-performance liquid chromatography (RP-HPLC). These purification methods are bioassay guided [52]. In general, bioassay-guided fractionation is utilized to screen and fractionate peptides. Confirmatory studies with synthetic peptides are required to identify the peptide(s) potentially associated with the observed biological activity. Several examples of marine bioactive peptides extracted by solvent fractionation are available. These include depsipeptides: geodiamolides H–N and P from marine sponge; cyclodepsipeptides: onchidin B from mollusk *Onchidium* species; and conotoxin GV from marine snail *Conus geographus* [52]. We provide some examples of the extraction of peptides in this review to give the readers an overall picture of the procedure of extraction based on the type of peptides extracted from the particular natural source.

Sruthy et al. [116] described a method that uses acetic acid-acetone for preliminary extraction. In this study, researchers isolated some antimicrobial peptides from the fish *Psenopsis cyanea* (Indian ruff). The fish organs, fish skin tissue, and gills were treated with acetic acid, and peptides were then precipitated using acetone. These peptides were further purified by solid-phase extraction and ion-exchange chromatography [116].

During the extraction and screening procedures, crude peptides can be extracted first and then screened for biological activity. If the crude extract exhibits any biological/pharmacological activity, then further purification can be carried out. This helps to avoid extensive and time-consuming steps involved in obtaining a final pure product that may not exhibit biological activity. Carstens et al. [77] describe the isolation of a disulfide bond containing peptides from a marine sponge. For the preliminary extraction of the crude peptides, a simple three-step extraction was used in which different proportions of acetonitrile and water mixture were incorporated [77]. Once the molecular ion of the peptide of interest was confirmed in the first stage of purification, size-exclusion and HPLC methods were used to purify it further. Previously, trichloroacetic acid (TCA) has been used for the preliminary extraction of peptides from marine sources [109], but in recent studies, it was found that trichloroacetic acid is not a good choice due to problems of protein/peptide precipitation and stability in acidic conditions. The acidic pH and negative charge of TCA responsible for the denaturation of some proteins lead to precipitation, and such protein precipitates are difficult to re-dissolve [117,118].

One method for the isolation and extraction of bioactive peptides from marine sources is hydrolysis of the parent protein with the help of proteolytic digestive enzymes. Enzymes from plant, bacterial, or fungal sources can also be used for hydrolysis. The optimization of reaction conditions is important for the expected enzyme activity. The reaction medium, pH, and temperature of the protein solution are some of the critical factors for optimization [119]. Ideal pH and temperature conditions for enzymes from different sources are listed in Table 2.

Ngo et al. [120] reported that optimum physiological conditions for enzymatic reactions and sequential enzymatic digestion using enzymes from microorganisms and the animal digestive system could be used to obtain several bioactive peptides. The degree of hydrolysis by enzymes is a critical parameter, having a direct effect on the size and sequence of the peptides as well as on the compositions of amino acids in the resultant peptides [94].

An example of enzyme hydrolysis of marine sources for generating bioactive peptides is provided by Li et al. [113], where an angiotensin-converting enzyme inhibitor was isolated. The idea was to use food proteins to extract bioactive peptides by enzyme hydrolysis. Marine fish are a rich source of proteins, and fish is one of the major types of seafood that can be utilized for pharmaceutical purposes. In this study, proteins obtained from the shellfish razor clam were hydrolysed by five different enzymes, namely, alcalase, pepsin, trypsin, flavourzyme, and *Actinomucor elegans* (fungi) extracted crude proteases. The authors focus on this shellfish in particular because of the large amount commonly obtained for consumption. It was estimated that nearly 786,000 tons of the cultured razor clam was harvested in 2014 [113]. To extract potential ACE inhibitors, different enzymes were used. For each enzyme, reaction conditions were optimized to yield specific peptide fragments that have ACE inhibitory activity. Studies showed that, among the enzymes, flavourzyme was the most efficient for hydrolysis (apart from crude proteases from the fungus *Actinomucor elegans*), followed by alcalase. After hydrolysis, the extracts obtained were exposed to gel filtration and reversed-phase HPLC, and amino acid sequences were analyzed by MALDI-TOF/TOF MS/MS [113]. Using an enzyme hydrolysis procedure, a novel ACE inhibitory peptide with an inhibitory activity IC_50_ of 9 μM was obtained.

These proteolytic enzymes from different sources can be used separately or in combination for a better and faster yield. Kumar et al. [121] used a novel combination of proteolytic digestive enzymes for the hydrolysis process. For the isolation of antioxidant peptides from two different fishes, they treated fish skin suspension with pepsin for 2 h, after which trypsin and α-chymotrypsin were added for a further 2.5 h incubation [121]. The enzymatic hydrolysis process for the production of peptides from parent proteins turns out to be the best method, as it is comparatively inexpensive, gives higher and faster yields, and produces more clean output that fits the requirements of industrial production [122].

Bioactive peptides are also extracted following bacterial fermentation of milk proteins where *Lactobacilli* is commonly used. Lactic acid bacteria are known to produce potent bioactive peptides from milk. However, microbial fermentation of meat and fish proteins is not viable because of the poor proteolytic activity of lactobacilli that is used in fermentation. As a result, the isolation and extraction of bioactive peptides by fermentation of marine sources are not very popular. In terms of safety, lactic acid bacteria have been considered safe in food and drink and are used as probiotics. The protease of lactic acid can hydrolyze various proteins to produce different oligopeptides that can be used in pharmaceutical applications. In fact, one potent ACE inhibitor was produced during milk fermentation by lactic acid bacteria [123,124]. Although this method is not popular, lactic acid fermentation has been used for the production of bioactive peptides in a few cases. In this method, the tissue or solid extract from the marine source is fermented with some microorganisms, resulting in the hydrolysis of proteins to yield peptide fragments [125].

Wang et al. [126] reported the production of a bioactive angiotensin-converting enzyme (ACE) inhibitor peptide obtained by the fermentation of marine shrimp, *Acetes chinensis*, with *Lactobacillus fermentum* [126]. Three ACE inhibitors produced by this method exhibited IC_50_ values in the 2–4 μM range. Although this is not a popular method of extraction of peptides from marine sources, authors have shown that fermentation is a simpler and cheaper method to produce peptides compared to protease degradation.

## 3. Classification of Marine Resources

**Figure 3:**
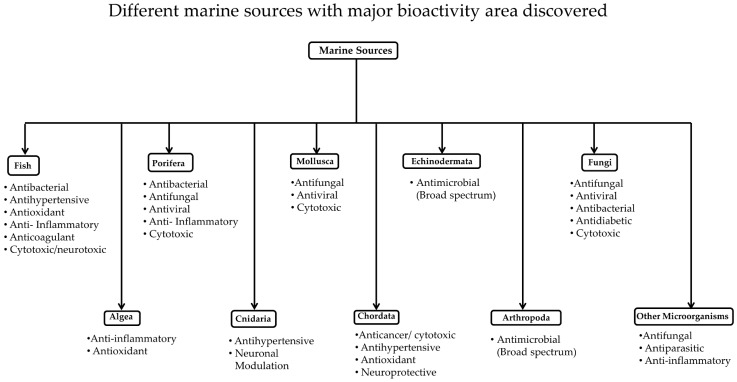
Classification of marine sources of peptides/peptidomimetics and their possible therapeutic applications.

Compounds derived from marine organisms have become a prolific source of drug discovery, but they are less known because of they are comparatively difficult to access and collect. Most studies are conducted with samples that can be collected near the shore. Marine peptides contribute 8% of the total bioactive marine compounds [127]. A high percentage of new compounds were isolated from a marine invertebrate, marine algae, and marine microorganisms. Among the invertebrates, most of the bioactive compounds were isolated from the phyla Porifera, Coelenterate, Mollusca, Chordata, and Echinodermata. Of these, about 75% of the bioactive compounds from invertebrates belong to the phyla Porifera and Coelenterate [128]. Most of the peptides from the phyla Porifera and Chordata are found to be cyclic peptides and depsipeptides (for example, homophymine A and didemnin B). Mollusca is also considered a source of cyclic peptides and depsipeptides, such as keenamides and kahalalides, along with linear peptides with disulfide bonds, such as conotoxins. Similarly, the phylum Cnidaria includes animals that produce linear toxins such as neurotoxin AV3 from the sea anemone *Anemonia viridis* [52,82]. Here, we discuss the studies related to peptides and peptidomimetics and polypeptides derived from marine sources based on the phylum from which they are derived (Figure 3). Compounds derived from the phyla Porifera, Cnidarian, Chordata, Echinodermata, as well as algae, fungi, and bacteria are described.

### 3.1. Marine Invertebrates

#### 3.1.1. Porifera

Sponges are the oldest metazoans; they are filter-feeding organisms found from the shore to deep water, and they are the major studied organisms in the phylum Porifera. Since 2010, 5300 natural products that contribute about 30% of the total known marine natural products have been isolated from marine sponges. Alkaloids, terpenoids, steroids, and peptides are known metabolites from sponges that provide resources for drug discovery [129]. Sponges contain cyclic peptides and some linear peptides composed of unusual amino acids, making sponges an interesting subject of study for pharmaceutical and biochemical research [130].

**Figure 4:**
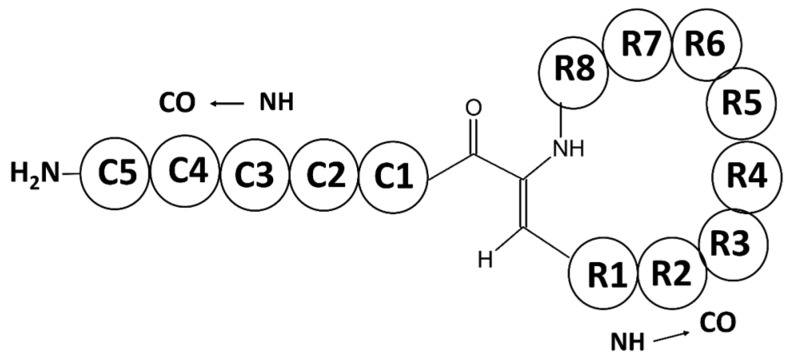
Callyaerin, a cyclic peptide derived from Indonesian sponge. Adapted and reproduced with permission from Elsevier, Ibrahim et al. *Bioorg. Med. Chem.* **2010**, *18*, 4947–4956.

**Figure 5:**
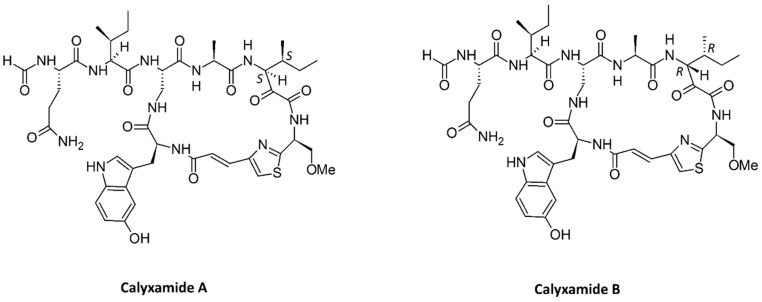
Peptides derived from the marine sponge *Discodermia calyx.* Reprinted with permission from Kimura et al. *J. Nat. Prod.* **2012**, *75*, 290–294. Copyright (2012) American Chemical Society.

Peptides derived from marine sponges have been evaluated in in vitro systems for their potential as therapeutic agents for many human diseases. The Indonesian marine sponge *Callyspongia aerizusa* was extracted with methanol, and the peptide fractions were separated using vacuum liquid chromatography with dichloromethane, ethanol, and methanol in the mobile phase. The ethanolic fraction yielded seven cytotoxic cyclic peptides, callyaerins A–G. These peptides contained a number of unusual prolines in their structure. Callyaerins A–F (Figure 4) showed antibacterial and cytotoxic activity in L5178Y, HeLa, and PC12 cell lines, and callyaerin H showed antifungal activity [130]. Along with known cyclic peptides with a linear side chain, such as callyaerins A–G from the same marine source, researchers found five new derivatives, namely, callyaerins I–M. These new peptides were assessed for antitubercular activity using resazurin dye reduction and cytotoxic activity against THP-1 (human acute monocytic leukemia) and MRC-5 (human fetal lung fibroblast) cell lines. Callyaerins A and B showed potent antitubercular activity, whereas callyaerin C showed mild antitubercular activity. Callyaerin A did not show cytotoxic activity in normal human cell lines, which suggests that it is a strong candidate for antitubercular application [70]. Cyclic peptides obtained from a methanolic extract of the marine sponge *Discodermia calyx*, collected in Japan, was further partitioned in hexane and ethanol. The ethanolic fraction produced calyxamides A and B (Figure 5), which showed potential cytotoxic properties against P388 murine leukemia cells with IC_50_ values of 3.9 and 0.9 μM, respectively [131].

**Figure 6:**
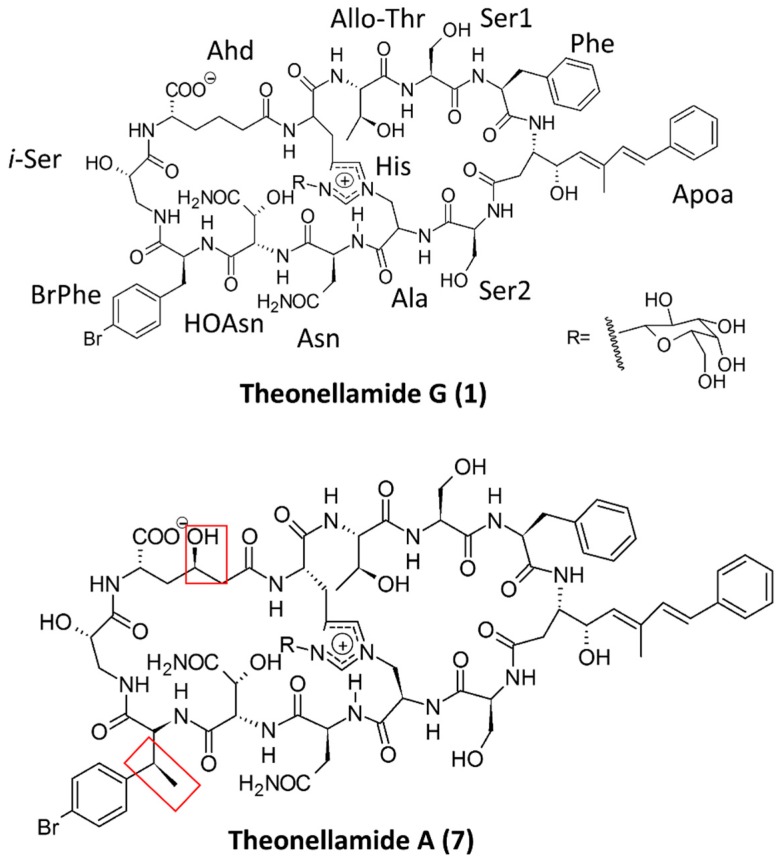
Antifungal and cytotoxic bicyclic dodecapeptides obtained from a marine sponge of *Theonella* species. Adapted from Youssef et al. *Mar. Drugs* **2014**, *12*, 1911–1923. Reprinted with permission from Matsunaga et al. *J. Org. Chem.* **1995**, *60*, 1177–1181. Copyright (1995) American Chemical Society.

Theonellamides (Figure 6) are antifungal and cytotoxic bicyclic dodecapeptides obtained from a marine sponge of *Theonella* species. Earlier reported butanol extracts of marine sponge *Theonella* species led to the isolation of bicyclic decapeptides theonellamide A–E, which had cytotoxic and antifungal properties. Further study of theonellamide-A showed that it binds with 3β-hydroxyl groups of sterols, cholesterol, and ergosterol found in yeast cells and causes membrane disruption and damage in yeasts, which was assessed by solid-state 31P NMR and confocal microscopy. Also, theonellamide-G showed potent antifungal activity against wild and amphotericin B-resistant strains of *Candida albicans* [74,75].

An Australian marine sponge, *Pipestela candelabra*, was successively subjected to extraction with hexane, dichloromethane, and methanol, followed by purification by preparative HPLC. For the elution of fractions, a C18 preparative column was used with a linear gradient from 100% water to 100% methanol (0.1% trifluoroacetic acid (TFA) in both solvents) for 60 min. In the second stage, a Hypersil BDS C18 column was used to separate the selected fractions and obtain two major classes of peptides known as hemiasterlins and milnamides. The structures of these peptides contain unnatural amino acids such as *tert*-leucine and *N*-methylvinylogous valine. A dipeptide fragment in the molecule is incorporated with tri- or tetra-methylated tryptophan to produce the hemiasterlins. They may also have a tetrahydro-β-carboline moiety in the molecule, that results in milnamides. Previously, the extraction of peptides from Australian sponge *Pipestela candelabra* had produced eight known compounds. Along with these known compounds, two new compounds were discovered. All these compounds were studied for their antiproliferative activity against human prostate cancer cells (PC3). To evaluate the selectivity of these compounds for prostate cancer cells, their activity was compared to human neonatal foreskin fibroblast non-cancer cells (NFF) by using cell proliferation assay. All compounds showed antiproliferative activity against PC3 cancer cell lines with IC_50_ values ranging from micromolar to nanomolar concentration, and they showed 2.6 to 8.6 times more selectivity for PC3 against the non-cancer cell line NFF. Milnamide A showed high potency (IC_50_ = 11 nM) as an antiproliferative agent for prostate cancer cells. Milnamides E–G exhibited potential activity, similar to that of milnamide A. The newly isolated compound, hemiasterlin D, showed 2.20 and 8.16 nM IC_50_ values in antiproliferative activity against PC3 and NFF cells, respectively [76].

**Figure 7:**
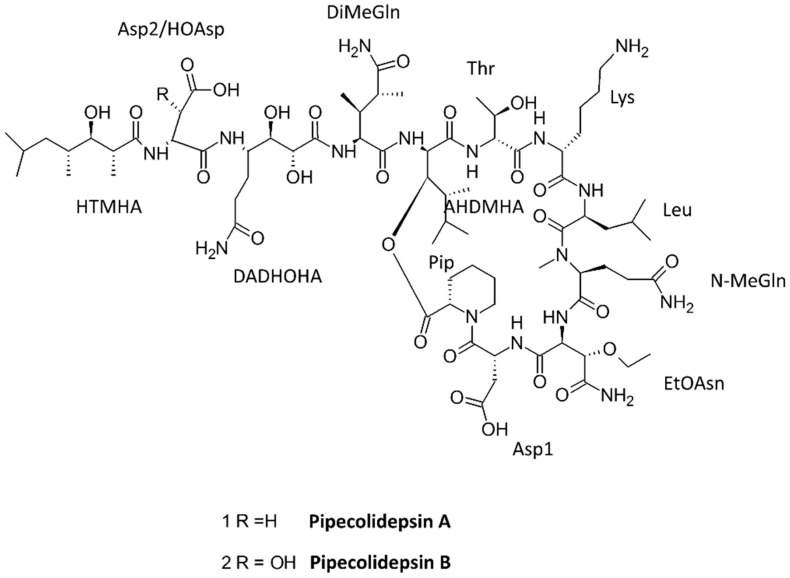
Pipecolidepsins A and B; cyclic depsipeptides identified from the extraction of the Madagascan Sponge *Homophymia lamellose.* Reprinted with permission from Coello et al. *J. Nat. Prod.* **2014**, *77*, 298–303. Copyright (2014) American Chemical Society.

Two new cyclic depsipeptides, pipecolidepsins A and B (Figure 7), were identified from the extraction of the Madagascan sponge *Homophymia lamellose* with 2-propanol, followed by partitioning with water, ethanol, and n-butanol. The extract was found to be cytotoxic against human cell lines of lung cancer (A-549), colon cancer (HT-29), and breast cancer (MDA-MB-231) because of the presence of these two bioactive peptides [73]. Similarly, an acetonitrile/water extract of the marine sponge *Geodia barretti* extracted with acetonitrile/water in different ratios led to the discovery of two peptides, barrettides A and B, having two disulfide bonds each. These peptides formed an unusual ladder-like structure with a disulfide bond and long β-hairpin structure. These two peptides showed an antifouling effect with barnacle larvae, but failed to show any antimicrobial effect in Gram-positive *Staphylococcus aureus* and Gram-negative *Escherichia coli* strains [77].

Sponges also produce peptides that have antiviral activity. Well-known peptides from a sponge that have antiviral activity are callipeltin A, neamphamide, papuamides A–D, and mirabamides A–D. Lu et al. [64] extracted the depsipeptides known as mirabamides E–H from the sponge *Stelletta clavosa*. These peptides have unusual structures with a rhamnose group, 2,3-diaminobutanoic acid, and a few modified amino acid residues in their structures. The difference in the structures of the four peptides is in the rhamnose unit. The four depsipeptides, isolated and purified, were able to inhibit the HIV-1 entry in an in vitro assay with one of these peptides exhibiting an IC_50_ of 40 nM. Plaza et al. [132] described the isolation of peptides from different deep-water specimens of *Theonella swinhoei* and *Theonella cupola* that have shown antiviral activity. One of them was a sulfated peptide named mutremdamide A, and the others were *N*-methylated peptides called koshikamides C–H. Among these, koshikamides C–E are linear undecapeptides, and F–H are 17-residue depsipeptides containing a 10-residue macrolactone. Antiviral activity assays suggested that cyclic peptides were active in inhibiting HIV-1 entry at low micromolar concentrations, while linear peptides were inactive. Some of these, as well as other peptides from marine organisms, are described in a recent review by da Mata et al. [133]. Theopapuamide B, a new depsipeptide from the Indonesian sponge *Siliquariaspongia mirabilis* was shown to have cytoprotective activity against HIV-1 in vitro [62,67]. Theopapuamides are peptides with unusual functional groups, 3-acetamido-2-aminopropanoic acid and 4-amino-2,3-dihydroxy-5-methylhexanoic acid, that also contain an *N*-terminal fatty acid, making them lipopeptides.

#### 3.1.2. Cnidaria

Cnidarians comprise thousands of species, including hydroids, jellyfish, anemones, and corals, which provide enormous resources for drug discovery. Some of these species are carnivorous, whereas some can perform photosynthesis as an alternate source of energy. They contain venom which helps them to capture their prey and to protect themselves, so cnidarians are considered to be toxic; they secrete toxins that interact with neuronal functions and have cytotoxic properties. Compounds were extracted from *Palythoa caribaeorum* by squeezing polyp tentacles in deionized water, and the solution was centrifuged to extract the venom, which was then evaluated for electrophysiological activity against voltage-gated sodium and calcium channels of rat superior cervical ganglion (SCG) neurons. The venom inactivated the sodium channel and blocked the potassium and calcium channels in rat SCG [79].

Collagen protein of the jellyfish *Rhopilema esculentum* was extracted, and the extract was subjected to hydrolysis with alcalase, producing jellyfish collagen peptides (JCPs). JCPs showed an antihypertensive effect [80]. Another ACE inhibitor peptide was obtained from the Filipino box jellyfish *Chiropsalmus quadrigatus*. Extraction and hydrolysis with pepsin and papain of the tentacles of the jellyfish provided bioactive peptides. From further bioassay-guided fractionation, purification, and sequence analysis, a peptide with the sequence Ala-Cys-Pro-Gly-Pro-Asn-Pro-Gly-Arg-Pro was found to show ACE inhibitory action in vitro with an IC_50_ value of 2.03 μM [81].

Salinamide A and B are bicyclic depsipeptides produced by *Streptomyces* species, a marine bacterium isolated from the surface of the jellyfish *Cassiopeia xamachana*. Both salinamide A and B exhibit antibacterial activity against Gram-positive as well as Gram-negative bacteria. The total synthesis of salinamide A has also been reported [134]. Although salinamide structures were discovered in early 1990, the molecular mechanism was not yet explained in detail. Sallinamide is known to inhibit RNA polymerase (RNAP). Degen et al. [135] elucidated the details of the structure and molecular mechanism of how salinamide binds to RNAP and inhibits RNA synthesis. Based on the details of the binding mechanism of salinamide A to RNAP, they propose that the binding site of salinamide is different from the binding site of other antibacterial drugs to RNAP. Further, when bacetria were treated with salinamide in combination with a current antibacterial drug that targets RNA polymerase, resistance to the antibacterial drug was not detected. Such studies that detail peptide-macromoleular complexes will be very useful in designing novel antibacterial drugs. A new salinamide F, which exhibits inhibition activity of RNAP, was also identified [136].

#### 3.1.3. Mollusca

Mollusca contains the second-largest number of species, including snails, tusk shells, octopus, sea hares, oysters, and clams. Because of their biodiversity, Mollusca produces peptides that are linear, cyclic, and conjugated. Some well-known examples of peptides from Mollusca are kahalalide F, dolastatin 10, ILX-651, and cemadotin; these are all well-characterized and some of them have already reached clinical trials for therapeutic purposes [137]. Cone snails use these peptides as a tool to paralyze and capture their prey. On average, each cone snail uses a cocktail of 100 peptides to synergistically paralyze its prey within seconds. Recent studies by Peng et al. [138], using genomic sequencing and electrospray ionization mass spectrometry, reported the presence of 1000 to 2000 peptides in a single cocktail of the sample [58,139]. More than 700 species of cone snail have been identified, and it is estimated that nearly 100,000 conopeptides may exist. These conopeptides are broadly classified into two groups; those with multiple disulfide bonds (*Conus* peptides, conotoxins) and those without. Conotoxins have 10 to 40 residues and contain two or three disulfide bonds. These conotoxins have proven to be a valuable asset for the study of neuroscience, as they block neuromuscular or nerve signaling [138,140]. Several conotoxins are in clinical trials or have reached the market. Ziconotide, a conotoxin derived from the marine cone snail *Conus magus*, is used as an analgesic. However, it has limited therapeutic application because of a lack of efficacy when delivered through oral or intravenous administration. There are attempts to improve the proteolytic stability of conotoxins [141,142]. Because of the diverse nature of their sequence, conotoxins target a variety of pharmacological receptors such as calcium channels, sodium channels, nicotinic acetylcholine receptors, noradrenaline transporters, *N*-methyl-d-aspartate (NMDA) receptors, and neurotensin receptors [143]. Structural details of conotoxins are well established. Some peptides extracted from cone snails are known to act as potent and specific antagonists of the *N*-methyl-d-aspartate receptor (NMDAR) [144,145]. These are known as “sleeper” peptides because they induce sleep. Franklin et al. [86] extracted peptides from the venom ducts of the Indian cone snail *Conus araneosus* by squeezing and extracting venom with 50% acetonitrile. After extracting the crude sample, it was subjected to RP-HPLC and mass chromatography, and 14 peptides were isolated. Among these, three peptide sequences, namely, ar3g, ar3h, and ar3i, exhibited posttranslational modifications. One of these peptides was evaluated for sleep-inducing activity in a mouse model [86].

As mentioned above, conotoxins can be used to identify specific pharmacological targets. Christensen et al. [146] identified a new antagonist of a particular type of acetylcholine receptor, α9α10 nAChR from the venom of *Conus geographus.* Conotoxin aS-GVIIIB was isolated from the crude venom of *Conus geographus*, extracted with water/acetonitrile/trifluoroacetic acid and purified using analytical HPLC. Structural characterization of the lead compound indicated that the peptide has a disulfide bond arrangement similar to that of the σ-conotoxin superfamily. Receptor selectivity studies of αS-GVIIIB suggested that the peptide was over 100-fold selective for the α9α10 nAChR compared to other nAChR subtypes. Cone snails also use insulin in their venom to capture their prey by rapidly inducing hypoglycemic shock. Insulin from *Conus geographus* G1 (Con-Ins G1) has similarities to human and fish insulin, but lacks the *C*-terminal segment of the B chain of human insulin. Smith et al. [147] elucidated the structural details of insulin from *Conus geographus* G1 and showed that Con-Ins G1 binds to the human insulin receptor and activates human insulin. Additionally, the peptide was found to be in monomeric form, acting as a mimetic of human insulin. Such structures can be used as templates for designing rapid-acting therapeutic insulin for diabetic patients. Characterizations of conotoxins and their pharmaceutical applications have also been described in review articles [54,148,149].

Invertebrates produce antimicrobial peptides that are useful in fighting infections that develop resistance. Because of their humoral immune response, they fight the infection in an efficient way. Most antimicrobial peptides derived from marine sources have an amphipathic character with many hydrophobic amino acid residues. Lopez et al. [85] described the extraction of a new peptide from the marine snail *Cenchritis muricatus.* Marine snails collected from Cuba were subjected to extraction through homogenization and centrifugation. The crude extract showed the potent antimicrobial effect and antifungal activity. The extract was subjected to digestion with trypsin, resulting in several hydrophilic peptides. The peptide Cm-p1 showed weak antimicrobial activity, but good antifungal activity [84,85].

#### 3.1.4. Chordata

**Figure 8:**
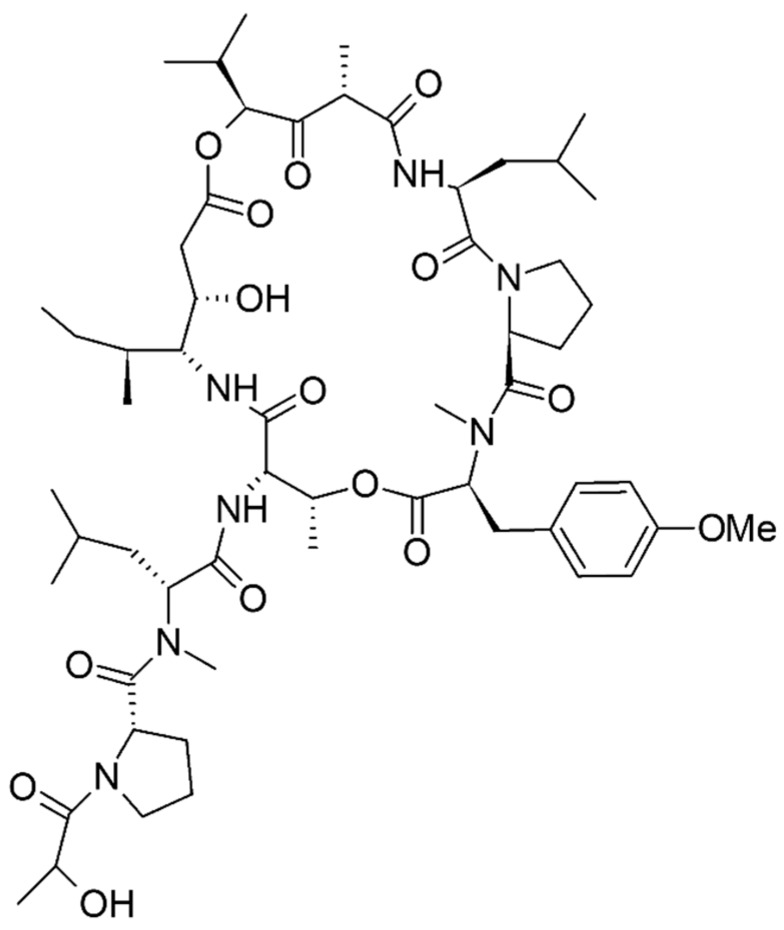
Cyclic depsipeptides known as didemnins identified from the extraction of the Caribbean tunicate *Trididemnum solidum.* Reprinted with permission from Xu et al. *J. Am. Chem. Soc.* **2012**, *134*, 8625–8632. Copyright (2012) American Chemical Society.

Tunicates are marine invertebrate animals that are marine filter feeders with a water-filled, sac-like body structure. Tunicates possess potentially useful compounds such as didemnins (Figure 8), which have anticancer, antiviral and immunosuppressant properties. Cyclic depsipeptides called didemnins have been obtained from the Caribbean tunicate *Trididemnum solidum*. More than nine didemnins (didemnins A–E, G, X and Y) have been isolated from the extract of *Trididemnum solidum*. Among these, didemnin B possesses the most potent biological activities and was the first marine peptide to be investigated against cancer in humans. Due to only moderate cytotoxic activity and potential toxicity, it failed to reach further stages. However, a compound closely related to didemnin B, dehydrodidemnin B (also known as aplidine), isolated from *Aplidium albicans*, was found to have increased potency against cancer cells and decreased toxicity. Currently, aplidine is undergoing clinical trials against multiple myeloma [150]. Many derivatives of didemnin are being studied and have been found to have potent antitumor, anti-infective, antiviral, and immunosuppressive qualities [151,152].

As described in this review, there are several peptides derived from marine sources that act as ACE inhibitors. In one of the purified fractions of an extract of the tunicate *Styela clava*, a peptide with the sequence Ala-His-Ile-Ile-Ile was found. This peptide was extracted from flesh tissue after hydrolysis by protease enzymes and fractionation through an ultrafiltration membrane. Ko et al. [153] investigated the vasorelaxation effect of this peptide. The peptide was found to show an antihypertensive effect in the animal model. The same group of researchers evaluated the potential anti-inflammatory effect of enzymatic hydrolysates from *Styela clava* flesh tissue. The anti-inflammatory effect was assessed via nitric oxide (NO) production in lipopolysaccharide (LPS)-induced RAW 264.7 cell lines (macrophages) and in an in vivo zebrafish model. Protamex hydrolysate was proven to have an anti-inflammatory effect through the inhibition of the production of nitric oxide by reducing the expression of inducible nitric oxide synthase (iNOS) and cyclooxygenase-2 (COX-2). It also reduced the levels of IL-1β, IL-6, and TNF-α without showing potential in vitro cytotoxic effects in human macrophages or in vivo cytotoxicity in a zebrafish model [154]. Furthermore, the purified peptide was found to exhibit glucose uptake stimulation in skeletal cells through the AMP-activated protein kinase (AMPK) pathway and glucose transporter type-4 (GLUT4) [88].

Several synthetic ACE inhibitors have been studied as potential antihypertensive agents, and ACE inhibitors are available on the market for antihypertension. However, synthetic drugs have certain side effects such as coughs, taste disturbances, and skin rashes [155,156]. Wijesekara et al. [157] reported that peptides were produced from the seaweed pipefish muscle protein using papain, alcalase, neutrase, pronase, pepsin, and trypsin. Among them, the alcalase hydrolysate exhibited the highest ACE-I inhibitory activity. The alcalase hydrolysate was separated into four fractions (Fr1, Fr2, Fr3, and Fr4) by fast protein liquid chromatography (FPLC). Fraction 3 was further fractionized into four parts. Fr3-II and Fr3-III, having the sequences Thr-Phe-Pro-His-Gly-Pro and His-Trp-Thr-Thr-Gln-Arg, respectively, showed potent angiotensin-I-converting enzyme inhibitory activity with no toxicity toward human cell lines [157]. Cell viability assay showed no cytotoxicity of alcalase hydrolysate on human lung fibroblasts cell line (MRC-5). These results suggest that peptides derived from seaweed pipefish can be developed as antihypertensive ingredients in functional foods.

A peptide extracted from the seahorse *Hippocampus trimaculatus* was used as a neuroprotective medium in in vitro studies as a potential therapeutic agent for Alzheimer’s disease (AD). *Hippocampus trimaculatus*-derived neuroprotective peptide (HTP-1) was produced by the hydrolysis of dried and powdered *Hippocampus*, which was then further purified. The sequence of the peptide was identified as Gly-Thr-Glu-Asp-Glu-Leu-Asp-Lys. To investigate the neuroprotective effect of HTP-1, an in vitro AD model approach with a co-culture of Aβ42-stimulated murine microglia BV2 cells and PC12 neuron cultures was used. The idea was to protect the neurons from toxicity produced during the development of AD. HTP-1 showed a neuroprotective effect in PC12 cell lines from amyloid-β42 (Aβ42)-induced toxicity to neurons with an 85.52 ± 2.22% survival rate. The peptide was not toxic to microglia BV2 cells [90,158]. The peptide also exhibited properties of recognized neuroprotective peptides, such as the presence of hydrophobic amino acid residues and polarizable amino acids at both ends for solubility and 3–15 amino acid residue length.

#### 3.1.5. Echinodermata

Echinoderms are a well-known phylum of diverse species that include sea lilies, starfish, sea urchins, sand dollars, and sea cucumbers. They are mainly divided into five groups: Crinoidea, Ophiuroidea, Asteroidea, Echinoidea, and Holothuroidea. Antimicrobial peptides centrocins 1 and 2 were isolated from extracts of the green sea urchin *Strongylocentrotus droebachiensis.* The centrocins contain a heavy chain (30 amino acids) and a light chain (12 amino acids) forming an intramolecular dimeric structure. These cationic peptides showed potent broad spectrum antibacterial activity [96].

Plancitoxin I is a protein derived from the venomous spine of the crown of thorns starfish *Acanthaster plansi* by homogenization, centrifugation, and chromatographic techniques. Plancitoxin exhibited cytotoxicity in human normal skin fibroblast cells and cancerous cells, malignant melanoma cells, prostate carcinoma cells, lung adenocarcinoma cells, and hepatocellular carcinoma cells. It was found to be most potent in human melanoma cells, as it increased lactate dehydrogenase (LDH) concentration, reactive oxygen products, and nitric oxide formation, caused a change in mitochondrial membrane potential, induced apoptosis, and inhibited cell migration. A possible mechanism of cell cytotoxicity was thought to be through MAP kinase and p38 pathway [95].

#### 3.1.6. Marine Arthropods

Marine arthropods consist of lobsters, crabs, horseshoe crabs, and skeleton shrimp. Horseshoe crabs produce relatively large amounts of tachyplesin and polyphemusin peptides. Polyphemusin II is an 18-amino acid residue peptide amide that showed anti-HIV-1 activity through its specific binding to a chemokine receptor CXCR4 [159,160], discovered nearly two decades ago. Peptides from shrimp have also been isolated and evaluated for anti-infective properties [161,162]. In fact, antimicrobial peptide penaeidins were first discovered in shrimp [163]. Rolland et al. [100] reported the characterization of Ls-Stylicin1, an antimicrobial peptide from the shrimp *Litopenaeus stylirostris.* Ls-Stylicin1 is a polypeptide with a proline-rich *N*-terminal region and a *C*-terminal region containing 13 cysteine residues. The peptide was shown to be active against pathogenic filamentous fungi. An et al. [161] reported a new subclass of peptides in this family. In addition to proline-rich and *C*-terminal cysteine residues, the new class contains a unique serine-rich region. This new class of peptides showed antibacterial activity to some Gram-positive and Gram-negative bacteria. Destoumieux et al. [164] investigated the defense mechanism of penaeidins and described the molecular mechanism of chitin binding penaeidins as antimicrobial agents. Penaeidins are active against both bacteria and filamentous fungi. Their antifungal activity is believed to be mediated by their ability to bind chitin. From these studies, it was revealed that penaeidins are synthesized and stored in shrimp haemocytes, and that the peptides are localized in granulocyte cytoplasmic granules. These are released only when the organism faces a microbial challenge. Such studies provide the details of molecular mechanisms needed for the synthesis of peptides as antimicrobial agents for defense mechanisms in invertebrates. Another 23 amino acid residue peptide named PvHCt was shown to participate in the penaeid shrimp defense mechanism. PvHCt is produced from the proteolytic cleavage of the *C*-terminal of a major protein of shrimp plasma (*Litopenaeus vannamei* hemocyanin) when the shrimp faces a microbial challenge. PvHCt is an anionic peptide that exhibits amphipathic α-helical structure. The peptide was shown to bind to the membrane and induce local membrane damage, resulting in fungal cell death [165].

#### 3.1.7. Algae, Fungi and Bacteria

**Figure 9:**
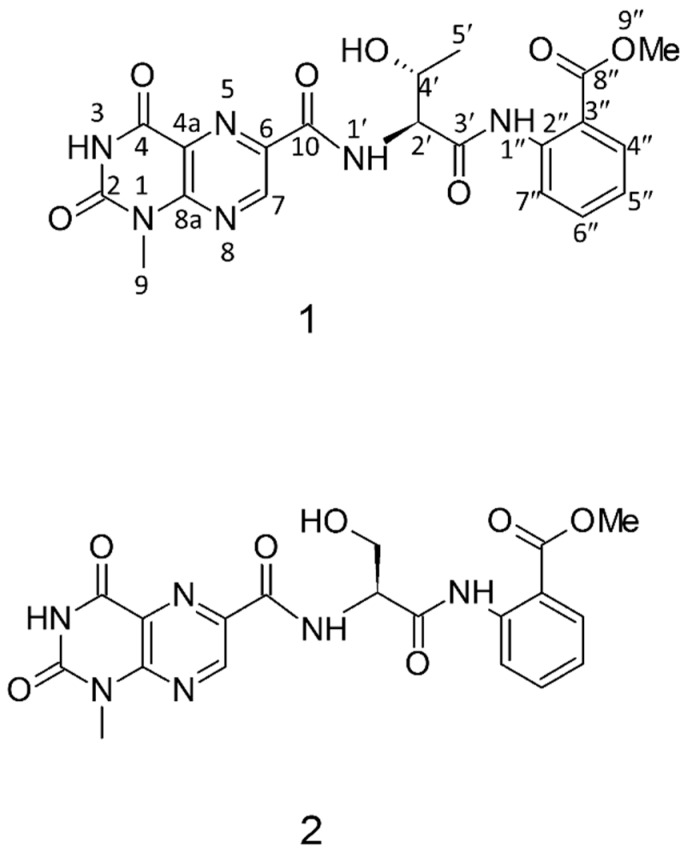
Peptidomimetics terrelumamides A (**1**) and B (**2**) isolated from the marine fungus *Aspergillus terreus.* Adapted from You et al. *Mar. Drugs* **2015**, *13*, 1290–1303.

Antimicrobial peptides (AMPs) exist in various species, including animals, plants, fungi, and bacteria, and act in the innate defense of the organisms or in microbial competitions. After the cultivation of the marine fungus *Aspergillus terreus,* the whole culture was subjected to extraction with methanol and further partition with water and n-butanol. Two peptides/peptidomimetics terrelumamide A and B were isolated from the marine fungus *Aspergillus terreus.* These peptides have a nonpeptide moiety in their structure, and usually have small amounts of amino acid residues in their sequences (Figure 9). They contain 1-methyllumazine-6-carboxylic acid, an amino acid residue, and anthranilic acid methyl ester connected by peptide bonds. These peptides were evaluated for antidiabetic and anticancer activities. The results suggested that terrelumamides seem to increase the sensitivity of insulin to the cells more effectively than glibenclamide, an antidiabetic drug. These peptides were also found to increase the level of adiponectin in an adipogenesis model of human bone marrow mesenchymal stem cells [107].

Fungi-derived cationic peptaibols are an important group constituting a large family of peptides that could be a potential source for new antimicrobial drugs [38]. Peptaibols [106,166,167] are characterized as linear peptides of 5–20 residues that contain a *C*-terminal amino alcohol, an acylated *N*-terminus, and a high proportion of non-standard amino acid residues, including α-amino isobutyric acid (Aib), isovaline (Iva), and hydroxyproline (Hyp). Trichokonin VI, a peptaibol produced by *Trichoderma pseudokoningii*, was extracted and evaluated for antibacterial activity. When Gram-positive bacteria were incubated with trichokonin VI, it showed concentration-dependent antibacterial activity to *B. subtilis.* A possible mechanism of antibacterial activity was proposed to be through the disruption of membrane permeability [168]. Isardins are cyclohexadepsipeptides that contain a β-alanine, an α-hydroxy acid, and four α-amino acids isolated from *Isaria* sp. and *B. felina* fungi. Du et al. [169] reported the isolation of three new isaridins, namely, isaridin G, desmethylisaridin G, and desmethylisaridin C1. The structures of these compounds were elucidated by the X-ray crystallographic method. Compound desmethylisaridin C1 exhibited antibacterial activity against *E. coli* with IC_50_ value of 8 μM.

Microbes are a potential source of new drugs. Metabolites of microbes can be used to study bioactivity. Most microbial products are cytotoxic and antimicrobial in nature. Two hexapeptides, JBIR-34 and JBIR-35, were isolated from a sponge-derived *Streptomyces* species using fermentation. These peptides were known to have antimicrobial and cytotoxic activity [102]. Mojavensin A, iso-C16 fengycin B, and anteiso-C17 fengycin B are three lipopeptides isolated from the marine bacteria *Bacillus mojavensis* B0621A through extraction by bioassay-guided fractionation and fermentation. Three peptides were found to show broad spectrum antifungal activity and cytotoxic activity against human leukemia cell lines [103]. Anti-inflammatory peptides containing γ-amino-acid and thalassospiramides A, D and G were obtained from the culture of bacterial *Thalassospira strain*, CNJ328. They inhibited the formation of nitric oxide in lipopolysaccharide-stimulated mouse macrophages without showing toxicity to human cells [104].

Antibacterial compounds were also discovered in bacterial species collected from sediments. Raju et al. [170] reported a new class of peptides; a glyco-hexadepsipeptide-polyketide from the marine-derived *Streptomyces* species isolated from sediment collected off South Molle Island, Queensland, Australia. The peptide exhibited inhibition of growth of Gram-positive and Gram-negative bacteria and drug-sensitive and multidrug-resistant clones of the malaria parasite *Plasmodium falciparum* with IC_50_ values in the nanomolar range. Furthermore, the peptide was less toxic to mammalian cell lines. Peptides that work on drug-resistant antimalarial strains will have high therapeutic value.

### 3.2. Proteobacteria

The most abundant marine bacteria phylum is Proteobacteria, which includes a wide variety of pathogens such as *Escherichia*, *Salmonella*, *Vibrio*, and *Helicobacter*. However, only a few bioactive compounds have been identified from this resource. The peptides identified from Proteobacteria are mostly non-ribosomal peptides (NRPs). Proteobacterial peptides are further classified as α-, γ- and δ-proteobacteria classes. A recent review by Desriac et al. [34] highlights some of the AMPs and includes a discussion of non-ribosomal peptides.

### 3.3. Actinobacteria

Peptidolipins are lipopeptides derived from a marine *Nocardia* species isolated from the ascidian *Trididemnum orbiculatum* [171]. Five new lipopeptides, peptidolipins B–F (1–5), were isolated, and each peptide contained a lipid chain. For peptidolipins E and F, an olefin and cyclopropyl group were present within the lipid chain. Among these peptidolipins, B and E exhibited moderate antibacterial activity.

## 4. Marine-Derived Products That Have In Vivo Efficacy

Research on marine organism-derived compounds started after the 1950s, when spongothymidine and spongouridine were isolated and identified from a marine sponge. This discovery led a new era in drug development, as compounds such as cytosine and adenine arabinoside were approved for anticancer therapy and antiviral therapy, respectively [172]. According to Tu (1974) [173], the study of marine peptides started in the 1960s with the investigation of sea snakes, and the first marine peptide reported was a neurotoxin. After the discovery of neurotoxins, the study of marine peptides grew immensely in the 1980s. This might be because of the advancement in the development of different isolation techniques and analytical instruments, such as NMR and HPLC [128]. More than 20,000 bioactive compounds have been isolated from marine organisms, of which bioactive marine peptides constitute more than 2000 [127]. Thus, there is a vast amount of literature related to the isolation of marine-derived peptides. However, most of the reports describe the isolation, purification, structural characterization, and in vitro activity of marine-derived peptides. There is very limited data on the in vivo efficacy of these peptides and very few marine-derived peptides have reached the pharmaceutical market. One of the reasons for the limited clinical use of these peptides is the high production cost of peptides. In particular, for antimicrobial peptides, the production cost of synthetic peptides is relatively high compared to that of conventional antibiotics [174,175]. However, efficient design of large-scale synthetic peptide reactors, bioreactors, and microwave-assisted synthesis has helped to produce peptides at a lower cost. Peptides produced by recombination methods by microorganisms have also brought down the production cost. Examples of this type of production include plectasin, the fungal defensing peptide produced in fungal expression system at high yields. Similar expression and production of protein systems have been adopted in the production of protein drugs [176]. In addition to this, proteins are produced using plant systems [177], and the same principles can be adopted in the production of peptide drugs [178,179]. Another major hurdle for peptide drugs is proteolytic degradation in vivo and rapid clearance. In order to show in vivo effect, the peptides must bypass in vivo hydrolysis and enzymatic degradation. However, many marine-derived peptides have cyclic structures and organic functional groups, making them relatively stable compared to linear peptides, which have natural amino acids in their sequence [180]. There are attempts to modify these marine-derived peptides using synthetic methods to incorporate biopharmaceutical properties and transform them into drug-like molecules. To improve the clinical efficacy of marine-derived peptides, new delivery systems have been formulated. In the following section, some of the recent studies related to in vivo pharmacological activity of marine drugs is described.

Zhuang et al. [80] isolated and purified collagen peptide, from alcalase hydrolysis of the jellyfish *Rhopilema esculentum*, which showed in vitro ACE inhibitory activity with an IC_50_ value of 43 µg/mL. The oral antihypertensive effect of the jellyfish collagen peptide (JCP) was determined in rats by inducing renovascular hypertension using the two-kidney one-clip model. Long term antihypertensive effect of the peptide was measured by administering the peptide through gastric intubation for 30 days. JCP could decrease the systolic and diastolic pressure, as well as decrease the heart rate of the hypertensive rats. JCP also decreased the level of angiotensin II in the kidney, which signifies that the antihypertensive effect shown is because of inhibition of ACE in the kidney.

Another antihypertensive peptide (Ala-His-Ile-Ile-Ile) was found through the enzymatic hydrolysate of the flesh tissue of the Asian tunicate, *Styela clava*. After in vitro evaluation of the peptide for ACE inhibitory effect, the peptide was incubated with isolated rat aorta, which showed the vasorelaxation effect on the isolated tissue. Vasorelaxation was observed to be blocked by the pretreatment of isolated aorta with nitric oxide synthase inhibitor. This study suggested the nitric oxide can be the target of the peptide. The efficacy of this peptide to exhibit antihypertensive effect in an animal model was measured by the oral administration of the peptide to rats. The antihypertensive effect was measured by observing the change in systolic blood pressure. The peptide could reduce the systolic blood pressure of rats, comparative to the activity of the well-known antihypertensive drug amlodipine [153].

Crude venom peptides from the Indian conus snail *Conus araneosus* were obtained by squeezing the venomous duct of snail. One of the peptides, named ar3j, showed a sleep-inducing effect in mice. After intraperitoneal injection of 2 nM of the peptide, it induced 2 h of sleep in mice, while 5 nM of the peptide induced 5 h of sleep, achieved without making the mice unconscious [86].

Zhu et al. [181] investigated the in vivo effect of oligopeptides from marine salmon skin (OMSS) to modulate type 2 diabetes mellitus (T2DM)-related hyperglycemia and β-cell apoptosis in rats subjected to a high fat diet. Based on this study, the authors concluded that treatment with OMSS significantly reduced the fasting blood glucose in diabetic rats. Marine collagen peptides (MCPs) from fish hydrolysate were used to evaluate the therapeutic effect in patients with type 2 diabetes mellitus (T2DM) in China [182]. The patients in the treatment group received an additional 13 g of MCPs daily for three months. Analysis of blood samples of patients suggested that there was a significant reduction in levels of fasting blood glucose, human glycated hemoglobin A1c, fasting blood insulin, total triglycerides, total cholesterol, low-density lipoprotein, and free-fatty acids in T2DM patients. However, there was an increase in the levels of insulin sensitivity index and high-density lipoproteins (HDL).

Many marine-derived peptides have shown antiproliferative activity against cancer cell lines, and a few of them have been evaluated for their ability to suppress tumor growth in an animal model [61]. Thiocoraline was isolated and purified from the marine bacterium *Verrucosispora* species, and is a symmetric octadepsipeptide with thioester and disulfide bonds in its backbone. The heterocylic chromophores help to intercalate with DNA (DNA bisintercalator), exhibiting antiproliferative activity. In addition, thiocoraline was shown to alter the neuroendocrine phenotype and activated the Notch pathway in medullary thyroid cancer (MTC) [183]. However, thiocoraline has poor pharmacokinetic properties and limited solubility. Recently, there have been attempts to increase the solubility of thiocoraline by synthesizing the analogs of thiocoraline [184]. Wyche et al. [183] developed a nanoparticle formulation of thiocoraline to overcome the solubility problem, and evaluated the in vivo efficacy of the formulation in a xenograft model of cancer, showing that thiocoraline was able to suppress the tumor growth in a mouse model.

Apratoxins are cytotoxic marine natural products produced by cyanobacteria. These natural products are known to modulate the co-translational translocation early in the secretory pathway, leading to receptor depletion at the surface of cancer cells. These apratoxins are known to downregulate the growth receptors and ligands for growth receptors that are key elements in cancer cell growth. Chen et al. studied the structure-activity relationship of apratoxins by total synthesis and alanine scanning, and designed a potent analog of apratoxin that exhibited in vivo efficacy in a colorectal tumor xenograft model [185].

Another peptide that is isolated from marine cyanobacterium *Symploca* species is largazole. This is a cyclodepsipeptide with a thioester moiety. Largazole acts like a prodrug, as it undergoes protein-assisted hydrolysis and rapidly liberates the bioactive species largazole thiol [186]. The thiol group binds to Zn^2+^ in the catalytic center of histone deacetylase, inhibiting the enzyme and ultimately leading to antitumor activity. Salvador et al. [186] designed analogs of largazole and improved its pharmacokinetic properties. They also evaluated the antitumor effect of largazole and largazole disulfide analogs using an HCT116 mouse xenograft model. Analogs of largazole exhibited better antitumor effect compared to the original largazole. This study is clear evidence of how one can improve the drug-like properties of marine-derived peptides using synthetic methods. In their study, Salvador et al. found that different analogs of largazole showed a similar biological activity profile in enzyme assays; however, differences were observed in cultured cells and in the mouse xenograft model depending on the modification of the compound using the prodrug strategy. Largazole is under preclinical evaluation for its anticancer properties. Other marine-derived peptides that are extensively studied for their anticancer effect are jaspamide and geodiamolide H. Jaspamide had hurdles to progress as a potential candidate for drug development because of its narrow margin of safety observed between doses required for efficacy in mouse tumor models, and severe acute toxicity in animal models [187].

Marine peptides that have shown anti-inflammatory properties have been evaluated for their pharmacological properties in animal models. Halipeptins have been isolated from the marine sponge *Haliclona* species. Halipeptins are 17-membered cyclic depsipeptides, while halipeptins A and B are mixed-biogenesis metabolites consisting of a peptidic portion connected to a polyketide framework containing 1,2-oxazetidine-4-methyl-4-carboxylic acid, 3-hydroxy-2,2,4-trimethyl-7-methoxydecanoic acid (HTMMD), and *N*-methyl-delta-hydroxyisoleucine; the HTMMD residue can also be substituted with 3-hydroxy-2,2,4-trimethyl-7-hydroxydecanoic acid [188]. The molecular target of halipeptins is not known. Randazzo et al. [188] evaluated the anti-inflammatory effect of halipeptins in mice models and found that halipeptin A exhibits very a potent anti-inflammatory effect; they compared its effect with that of naproxen and indomethacin. Compared to the anti-inflammatory drugs indomethacin and naproxen, halipeptin A was 40 and 130 times more potent under the experimental conditions they studied in animal models. A recent review by Rangel et al. [61] provides an overview of marine-derived depsipeptides that have shown potent in vivo activity. There were many failures as well as success stories of marine-derived peptides, in particular, antimicrobial peptides. Some of these peptides are effective in vitro but exhibit toxicity. A review by Giuliani et al. highlights some of the antimicrobial peptides that went on to later stages of drug development, but failed to make it to the market [175].

## 5. Drugs on the Market and in Clinical Trials

**Figure 10:**
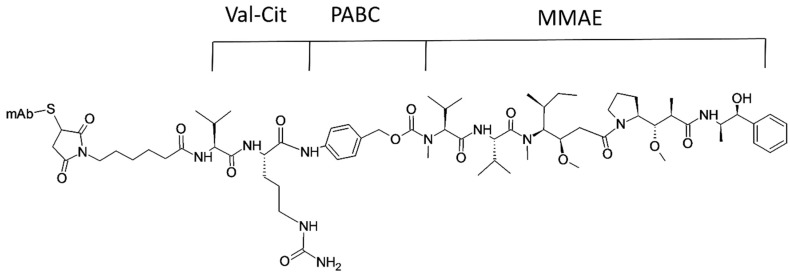
Structure of brentuximab vedotin. Adapted and reproduced with permission from Peter D Senter, Eric L Sievers. *Nat. Biotechnol.* **2012**, *30*, 631–637. Copyright (2012) Nature Publishing Group. mAb, monoclonal antibody; Val-Cit, Valine-Citrulline (linker); PABC, *p*-aminobenzyloxycarbonyl (spacer); MMAE, Monomethylauristatin E (a synthetic antineoplastic agent).

Many marine peptide drugs have reached the clinical trial phase (Table 3) and some of them, such as Ziconotide and Brentuximab vedotin (Figure 10), have been approved by the FDA and are in use in clinics. Although they exhibit higher target selectivity and potency, many peptides could not reach clinical studies. Low physical, chemical, and enzymatic stability, short half-life, and low oral bioavailability are the major reasons for the failure of most peptides [1]. Some of the marine peptides and their derivatives that made their way to clinical trials are discussed below.

Ziconotide, which is isolated from the fish-eating marine cone snail *Conus magus* and is available in the US market under the name Prialt, is an analgesic drug that acts by antagonizing N-type calcium channels. It is obtained using a synthetic method. This drug can be potentially beneficial for chronic pain as a non-opioid analgesic [189]. Derivatives of conotoxins are also being studied in clinical trials. XEN-2174, which is a derivative of conotoxin MrIA, is in clinical trial phase II as a noncompetitive inhibitor of norepinephrine transporters. Leconotide, which is a derivative of the drug Ziconotide, is in clinical trial phase I for the treatment of pain associated with cancer. Leconotide is less toxic and can be administered intravenously [198]. Many of the marine-derived peptides that are on the market or in clinical trials are discussed in a recent review by Cheung et al. [37]

Dolastatin 10, an anti-microtubule peptide derived from the marine mollusk *Dolabella auricularia*, reached clinical trial phase II, but because of its lesser effect in metastatic breast cancer, further studies were not completed. Many synthetic analogues of dolastatin 10 are being studied for their anticancer activity and are in different phases of clinical trials. Glembatumumab Vedotin is in clinical trial phases I/II for the treatment of breast cancer. It is a monoclonal antibody-conjugated derivative of dolastatin 10 that acts by binding to glycoprotein non-metastatic B (NMB). TZT-1027, which acts by binding with microtubules, is in clinical trial phase II for soft tissue sarcoma [194,195,202]. Cemadotin is another analog of dolastatin 10 that reached clinical trial phase I, but was withdrawn because of its cardiovascular toxicity and myelotoxicity [203].

Similarly, brentuximab vedotin, known as Adcetris in the US market, is a synthetic derivative of marine peptide dolastatin 10 conjugated to a monoclonal antibody. It is approved by the FDA for the treatment of classical Hodgkin’s lymphoma and systemic anaplastic large cell lymphoma. Brentuximab vedotin acts by targeting CD30 protein, which is expressed in large quantities in the target cancer cells [37].

**Figure 11:**
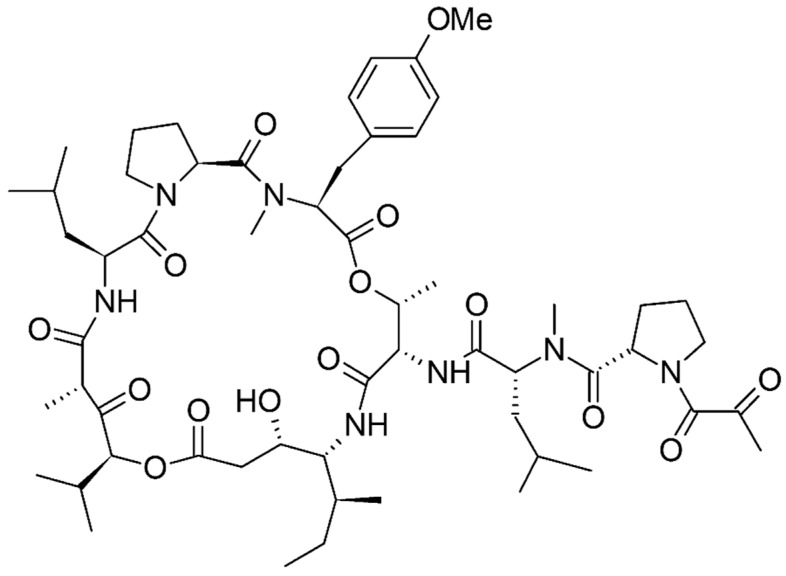
Derivative of didemnin known as Aplidine (Plitidepsin). Reprinted with permission from Adrio et al. *J. Org. Chem.* **2007**, *72*, 5129–5138. Copyright (2007) American Chemical Society.

Didemnin B, the first marine peptide to reach human clinical trials, is classified as an anticancer agent, and was originally derived from the tunicate *Trididemnum solidum*. For various toxicological reasons, it could not make its way toward further studies. Another derivative of didemnin known as Aplidine (plitidepsin) or dehydrodidemnin B reached clinical trials. Plitidepsin (Figure 11), in combination with Bortezomib and dexamethasone, is being studied for the treatment of multiple myeloma. According to clinicaltrials.gov, study ID NCT01149681, it is also being studied for the treatment of patients with primary myelofibrosis [151,152].

Tetrodotoxin, a potent toxin that has caused many human intoxications, was found in marine pufferfish *Tetraodontidae* species. Later, it was found in various sources, including marine bacteria and terrestrial organisms. It blocks the voltage-gated sodium channel and produces analgesic activity. Tetrodotoxin is in clinical trial phase III and II for the treatment of pain reduction in cancer and neuropathic pain, respectively [204].

Kahalalide F, a potential anticancer agent from the mollusk *Elysia rufescens*, and its diet green algae *Bryopsis* species, reached clinical trial phase II. However, because of lack of desired response in humans, it did not continue to further studies [191]. Possible synthetic derivatives of it are being studied to increase its potency. Elisidepsin (PM02734), a synthetic derivative of Kahalalide F, has completed a phase I clinical trial for the treatment of advanced malignant solid tumors and is in the process of phase II clinical trials. Elisidepsin was shown to insert into the plasma membrane of cancer cells and reorganize itself in the membrane; this caused the disruption of the plasma membrane and resulted in a loss of integrity of the membrane, causing necrotic death of cells [192,205]. Molina-Guijarro et al. [206] elucidated the detailed mechanism of loss of membrane integrity by elisidepsin and identified that glycosylceramides act as membrane targets of elisidepsin. Glycosylceramides in cells facilitates the insertion of elisidepsin, resulting in membrane destabilization and cell death. Cruz et al. [207] reported the synthesis of analogs of kahalalide F with the substitution of key amino acid residues that were responsible for the antitumor activity of the peptide. The overall charge and hydrophobicity of the peptide were changed by the substitution of functional groups in the peptide; however, the cyclic part of the peptide was not changed. The resulting peptide exhibited leishmanicidal activity at low concentrations. Based on the structure-activity relationship, the authors concluded that a net cationic character is necessary for leishmanicidal activity.

HTI-286 is a derivative of marine tripeptide hemiasterlin, which is derived from the sponge *Hemiasterella minor*. HTI-286 is an anti-microtubule, anticancer agent that acts by depolymerization of microtubules; it is in a preclinical study. Another derivative of hemiasterlin, peptide E7974, was found to be effective in a phase I study of colorectal, prostate, and larynx carcinomas. It acts by a similar mechanism, and is on its way to a phase II study [137,196].

## 6. Conclusions

Overall, marine resources provide an abundant source for peptide extraction. These peptides have a wide variety of applications from pharmaceuticals to nutraceuticals. With the advancement of technology and available devices to search for marine sources, more peptides will be discovered. Apart from this, improvement in the sensitivity of analytical techniques makes it possible to obtain pure peptides from natural resources. Once the peptides obtained from marine sources are characterized, a synthesis scheme can be developed to produce these peptides on a large scale for pharmaceutical applications. Furthermore, some of these active peptides can be modified for i.v. or oral administration. Thus, marine sources can serve not only as a rich source of food, but also as a valuable resource for nutrition, pharmaceuticals, and nutraceuticals. There are still some peptides in the deep, dark ocean waiting to be discovered.

## Figures and Tables

**Table 1:** Peptides derived from marine resources with possible therapeutic applications.

Peptide	Number of Amino Acids/Unnatural Amino Acids/Molecular Weight	Marine Source	Possible Application	Reference
**Porifera**				
Cyclic depsipeptides Mirabamides	3 N, 8 UNA	Sponge *Stelletta clavosa*	Anti-HIV and antibacterial	[63,64]
Papuamides A–F	11 UNA	Sponges *Theonella mirabilis* and *Theonella swinhoei*	Anti-HIV and cytotoxic	[65,66]
Celebesides A–C and	5 UNA	Sponge *Siliquariaspongia mirabilis*	Anti-HIV, cytotoxic, and antifungal	[67]
Theopapuamides B–D	2 N, 9 UNA	Sponge *Siliquariaspongia mirabilis*	Anti-HIV, cytotoxic, and antifungal	[67]
Callipeltins	5 N, 5 UNA	Sponge *Callipelta* sp.	Anti-HIV	[68,69]
Callyaerins A–G, I–M	9-12 N	Sponge *Callyspongia aerizusa*	Antimicrobial, antitubercular and antiviral	[70]
Discodermin A	11 N, 3UNA	Sponge *Discodermia kiiensis,*	Broad spectrum antifungal and antibacterial, inhibited starfish embryo development	[71]
Theonellamide G	7 N, 5 UNA	Red Sea sponge *Theonella swinhoei*	Antifungal and cytotoxic	[72]
Pipecolidepsins A and B	3 N, 8 UNA	Sponge *Homophymia lamellose*	Cytotoxic	[73]
Theonellamide A–E, G	7 N, 5 UNA	Sponge *Theonella* sp.	Cytotoxic and antifungal	[74,75]
Milnamide A	3 UNA	Sponge *Pipestela candelabra*	Antiproliferative and antitumor	[76]
Barrettides A and B	31 N	Sponge *Geodia barrette*	Antifouling effect with barnacle larvae	[77]
Koshikamides F–H	4N, 13 UNA	Sponges *Theonella swinhoei* and *Theonella cupola*	Anti-HIV	[78]
**Cnidaria**				
Toxin of *Palythoa caribaeorum*	1800–9000 Da peptides	Zoanthid *Palythoa caribaeorum*	Neuronal function modulation	[79]
Jellyfish collagen peptides (JCP)	200–600 Da	Jellyfish *Rhopilema esculentum*	Antihypertensive	[80]
Peptide Ala-Cys-Pro-Gly-Pro-Asn-Pro-Gly-Arg-Pro	10 N	Box jellyfish *Chiropsalmus quadrigatus*	Antihypertensive	[81]
Neurotoxin AV3	27 N	Sea anemone *Anemonia viridis*	Modulation of voltage gated sodium channel	[82]
**Molluska**				
Kahalalide F Analogs	8 N, 5 UNA	Mollusks *Sacoglossan* sp.	Antitumor and antifungal	[83]
Cm-p1 and Cm-p5	10 N	Marine snail *Cenchritis muricatus*	Antifungal	[84,85]
Crude peptide extract		Cone snail *Conus araneosus*	Sleep inductive	[86]
Hemocyanin 1 and 2	38 and 24 N	Sea snail *Rapana thomasiana*	Antiviral against herpes simplex virus (HSV)	[59]
Neurotensin (NT) and Contulakin-G	13 N in NT, and 15 N and 1 UNA in contulakin-G	Cone snail *Conus geographus*	Analgesic	[87]
**Chordata**				
Peptide (Ala-His-Ile-Ile-Ile, MW: 565.3 Da)	5 N	Tunicate *Styela clava*	Antihypertensive, antioxidant, cytotoxic, hepatoprotective	[88]
PC-1, PC-2 and PC-3	5 N	Large yellow croaker *Pseudosciaena crocea*	Antioxidant	[89]
HTP-1	8 N	Seahorse *Hippocampus trimaculatus*	Neuroprotective	[90]
EcDefensin	63 N	Estuary cod *Epinephelus coioides*	Antiviral against Singapore grouper iridovirus and viral nervous necrosis virus	[91]
Trunkamide A	3 N and 4 UNA	Ascidian *Lissoclinum patella*	Antitumor	[92]
SP-A and SP-B	9 N	Skate *Raja porosa*	Antioxidant	[93]
Hydrolysate fractions FrA3 and FrB2	585.91 and 528.78 Da peptides	Skipjack tuna *Katsuwonus pelamis*	Antioxidant	[94]
**Echinodermata**				
Plancitoxin I	358 N	Spine of crown of thorns starfish, *Acanthaster plansi*	Cytotoxic and apoptotic	[95]
Centrocins 1 and 2	119 and 118 N	Green sea urchin *Strongylocentrotus droebachiensis*	Broad spectrum antibacterial	[96]
**Arthopoda**				
SpHyastatin	135 N	Mud crab *Scylla paramamosain*	Antibacterial	[97]
Scygonadin	102 N	Mud crab *Scylla paramamosain*	Antibacterial and immune booster, antiviral against white spot syndrome virus	[59]
Histone H2A derivative sphistin	38 N	Mud crab *Scylla paramamosain*	Antimicrobial	[98]
Shrimp anti-lipopolysaccharide factor (SALF)	24 N	Shrimp *Penaeus monodon*	Increased expression of immune cells in mice, can be used as adjuvant in cancer vaccine, antibacterial	[99]
Ls-Stylicin1	82 N	Shrimp *Litopenaeus stylirostris*	Antibacterial, antifungal	[100]
**Algae, fungi and bacteria**				
Cyclic lipopeptides, Maribasins A and B	7 N and 1 UNA	Fermentation broth of the marine microorganisms *Bacillus marinus* B-9987	Broad spectrum antifungal	[101]
JBIR-34 and JBIR-35	4 UNA	Sponge derived actinomycete *Streptomyces* sp.	Antibacterial, cytotoxic	[102]
Mojavensin A, iso-C16 fengycin B, and anteiso-C17 fengycin B	7 N and 1 UNA	Bacterium *Bacillus mojavensis* B0621A	Cytotoxic, antifungal	[103]
Thalassospiramides A, D and G	(1, 4 and 2 NA) and (6, 3 and 5 UNA)	Bacteria *Thalassospira* strain CNJ328	Anti-inflammatory	[104]
Hormaomycins B and C	2 N and 6 UNA	Mudflat-derived *Streptomyces* sp.	Antibacterial	[105]
Aminolipopeptides Trichoderins A, A1 and B	2 N and 6 UNA	Marine sponge-derived fungus of *Trichoderma* sp.	Antimycobacterial activity against *Mycobacterium smegmatis, Mycobacterium bovis,* and *Mycobacterium tuberculosis*	[106]
Terrelumamides A and B	1 N	Marine fungi *Aspergillus terreus*	Antidiabetic (increased insulin sensitivity)	[107]
PPY1	5 N	Algae *Pyropia yezoensis*	Anti-inflammatory	[108]

N, Natural amino acids; UNA, unnatural amino acids.

**Table 2:** Optimal conditions for different proteolytic enzymes for hydrolysis reaction [93,94,113].

Major Source	Proteolytic Enzyme	pH	Temperature (°C)
Animals	Trypsin	8.0	37
	Pepsin	2.0	37
	α-Chymotrypsin	8.0	37
Plants	Papain	6.5	50
Microorganisms	Alcalase	9.5	50
	Neutrase	7.0	60
	Flavourzyme	7.0	55
	Protamex	6.0	40
	Kojizyme	6.0	40

**Table 3:** Marine-derived peptides that are on the market or in clinical trial.

Marine Peptides in Clinical Trial and on the Market	Marine Source	Therapeutic Application	Reference
Ziconotide	Marine cone snail *Conus magus*	Analgesic drug (FDA-approved)	[189]
Brentuximab Vedotin	Marine mollusk *Dolabella auricularia* Cyanobacteria *Symploca* sp. (Antibody-peptide conjugate)	Anticancer (FDA-approved)	[190]
Kahalalide F	Mollusk *Elysia rufescens*, and its diet green algae *Bryopsis* sp.	Anticancer (phase I clinical study)	[191]
Elisidepsin (PM02734)	Synthetic analogue of kahalalide F	Malignant solid tumors (phase I clinical study)	[192]
Tasidotin (ILX-651)	Dolastatin 15 synthetic analogues, marine mollusk *Dolabella auricularia*	Solid tumors, microtubule assembly, lung cancer; phase III clinical study, trials under review	[193]
Glembatumumab Vedotin	Derivative of dolastatin 10, marine mollusk *Dolabella auricularia* (Antibody-peptide conjugate)	Phase I/II for treatment of breast cancer	[194]
Soblidotin (TZT-1027)	Derivative of dolastatin 10, marine mollusk *Dolabella auricularia*	Phase II clinical trial for soft tissue sarcoma	[195]
E7974	Derivative of hemiasterlin, sponge *Hemiasterella minor*	Phase I study of colorectal, prostate, and larynx carcinomas; recommended for phase II	[196]
HTI-286	Derivative of hemiasterlin, sponge *Hemiasterella minor*	Metastatic prostate cancer (preclinical study)	[137,197]
XEN-2174	Derivative of ziconotide, marine cone snail *Conus magus*	Analgesic (phase I-II open label study)	[198,199]
Plitidepsin	Ascidian *Aplidium albicans*	Anticancer (phase I/II clinical study)	[200,201]
